# Descriptive Analysis of International Bariatric Surgery Tourism Services

**DOI:** 10.1007/s11695-023-06522-5

**Published:** 2023-02-24

**Authors:** Ariffin Azlan, Francis M. Finucane, Gerard T. Flaherty

**Affiliations:** 1 Bariatric Medicine Service, Centre for Diabetes, Endocrinology and Metabolism, Galway University Hospitals, Galway, Ireland; 2 School of Medicine, University of Galway, Galway, Ireland; 3 National Institute for Prevention and Cardiovascular Health, Galway, Ireland

**Keywords:** Obesity, Bariatric, Medical tourism, Surgical tourism, Health information

## Abstract

Inadequate access to public bariatric surgical services has favoured the growth of bariatric tourism. This study analysed data extracted from bariatric surgical centres that care for patients travelling from abroad. The research highlights apparent deficits in accreditation, communication, perioperative care, and travel health advice. An international registry of accredited bariatric tourism providers and patient education may be indicated.

## Introduction

Travel health risks in international travellers with obesity have only recently been described [1, 2]. Bariatric surgery is an effective weight loss intervention in patients with obesity. An unmet need for bariatric surgery in many countries has stimulated the growth of bariatric tourism worldwide [3], whereby patients with severe obesity make their own arrangements for travel to a bariatric surgical service in a different country or jurisdiction to their own. This is usually self-funded and initiated by the patient rather than by their general practitioner or physician. The lack of affordable bariatric healthcare and excessively long patient waiting lists are among the principal motivations for choosing bariatric tourism [4]. We previously identified barriers to accessing bariatric surgical care abroad in patients attending a bariatric medicine centre in Ireland. [5]

Attention to pre-travel health care and post-travel medical follow-up are crucial elements of a comprehensive medical tourism service. Lack of follow-up patient care is of particular concern among surgical tourists. A case was reported of erosion of a retained band following removal of an infected port in a 48-year-old female patient who travelled to Mexico for laparoscopic adjustable gastric banding [6]. The authors emphasise the need for appropriate patient follow-up and early recognition of procedural complications in bariatric tourists.

It is important that information available on clinic websites is comprehensive, accurate, transparent, and does not mislead. Previous analyses of medical tourism websites have exposed shortcomings in the online guidance provided to potential patients, such as in stem cell therapy [7]. We aimed to characterise the descriptions online of clinical services available to patients with obesity seeking bariatric surgical care abroad.

## Methods

### Study Design

A cross-sectional study of English language websites relating to bariatric surgical services which provide clinical care to self-referring patients from outside of their jurisdiction was conducted. Websites were scrutinised between March and July 2022 using relevant search terms on the Google® search engine.

### Variables

Data regarding clinic location, structure and accreditation, range and cost of procedures, surgical volumes, multidisciplinary assessment, perioperative care, clinical outcomes, patient follow-up, and travel health advice, including medical fitness to fly considerations were recorded. Information regarding promotional strategies, such as incentives, social media, third-party rating websites, and client testimonials, were also recorded. Duplicate websites were excluded from our analysis.

### Statistical Analysis

Data were analysed descriptively using frequencies and percentages.

## Results

Of 34 centres identified, the largest group were Eastern European (38.2%, *n* = 13) with the largest number of clinics globally located in Poland, Turkey, and Mexico (*n* = 4 each). Three centres were described as “centres of excellence”. Evidence of international accreditation was provided by 32.4% (*n* = 11) of facilities, but Joint Commission International accreditation was specified by only two centres. International expert guidelines for bariatric care (IFSO and NICE) were quoted by three units. Surgery volume was disclosed by 47.1% (*n* = 16) of websites, with procedure volumes ranging from 1200 to “over 7000”. The majority of clinics (82.4%, *n* = 28) mentioned the number of surgeons employed, with 44.1% of clinics (*n* = 15) having at least two surgeons on their staff.

Qualifying body mass indices (BMI) were specified by 79.4% (*n* = 27) of clinics, with BMI ranging from a minimum of 30 kg/m^2^ for gastric sleeve surgery without comorbidities to over 40 kg/m^2^ with comorbidity for a duodenal switch procedure. Comorbidities were mentioned in 11.8% (*n* = 4) of websites. One clinic specified an age range of 18 to 65 years for patient candidates. Costs varied widely for different procedures and across clinics, with price ranges of USD 3300–6300 (EUR 3123–5973) for gastric bands and USD 5900–16,000 (EUR 5594–15,170) for gastric bypasses. The benefits and risks of bariatric procedures were addressed by 55.9% (*n* = 19) of units. A single centre provided clinical outcome data and reported a 3% complication rate. Intensive Care Unit facilities were mentioned by 17.6% of bariatric centres (*n* = 6).

In-person or virtual consultations were recommended by 32.4% of clinics (*n* = 11). Live chat communication was offered by 20.6% of clinic websites (*n* = 7). Online testimonials featured in 96.7% (*n* = 29) of websites, with a single negative report published on one website. Third-party rating websites, predominantly Trustpilot®, were cited by 35.3% of centres (*n* = 12). Facebook was the most frequently used social media (70.6%, *n* = 24), followed by YouTube (50%, *n* = 17) and WhatsApp (29.4%, *n* = 10).

Liaison with the patient’s primary care physician was recommended by 23.5% (*n* = 8) of clinics. Few services referred to psychological (23.5%, *n* = 8) or dietetic (32.4%, *n* = 11) assessments and nurses were mentioned in 41.2% (*n* = 14) of websites. Most centres offered tourism packages and incentives, including ‘early bird’ discounts, free airport transfers, and discounted plastic surgery procedures (e.g. abdominoplasty). Very few websites provided specific travel health advice, with COVID precautions given by 29.4% of centres (*n* = 10), and fitness to fly and thromboprophylaxis discussed by 8.8% (*n* = 3) of websites each. Website information relating to postoperative patient care protocols are summarised in Table 1.

**Table 1:** Bariatric tourist follow-up care protocols

Measure	Proportion of clinics (*n*)	Comments
Postoperative recovery	70.6% (24)	Ranged from 1 to 5 days, with hotel recuperation mentioned by 7 clinics
Planned follow-up	67.7% (23)	Follow-up mostly restricted to surgical review, with limited involvement of other multidisciplinary team members
Provision of medical notes to patient	2.9% (1)	-
Provision of discharge summary to patient	14.7% (5)	Translated notes were available in one centre
Liaison with primary care physician	5.9% (2)	-

## Discussion

The subset of people living with obesity who travel across international borders to undergo bariatric surgery represents a vulnerable and understudied group of travellers. This is one of the first reported descriptive analyses of bariatric tourism services and the first to examine website content from a diverse range of international bariatric centres. It reveals deficits in online information regarding institutional accreditation, doctor-patient communication, perioperative care, and pre-travel health advice. A survey of bariatric surgeons found that medical tourists accounted for at least 2% of global bariatric procedures [4]. Mexico, Lebanon, and Romania emerged as leading bariatric tourism destinations, attracting patients with obesity mainly from the USA, the UK, and Germany.

In the absence of published standards for high-quality bariatric tourism information, it is important that bariatric centres provide clear, accessible, and transparent information on their websites to enable bariatric tourists to make informed decisions about travel for obesity surgery. Our findings highlight the significant variation in publicly available online information regarding surgical volumes, qualifying BMI criteria, financial costs, and surgical procedures among bariatric tourism providers. The lack of information on clinical outcomes and on the potential complications of bariatric surgery are potentially problematic.

Modern bariatric care is delivered by multidisciplinary teams, which are actively involved throughout the bariatric patient’s individualised care pathway. This study exposes potential deficits in online information regarding the multidisciplinary assessment and care of patients, including psychological and dietetic assessment. A lack of emphasis on follow-up with the patient’s general practitioner could contribute to poor patient outcomes and confound efforts to manage complications arising postoperatively in the patient’s home country.

The almost complete absence of general or country-specific pre-travel health advice was notable, given that there is guidance in the medical literature about travel with obesity [2], and recommendations for providers of medical and surgical tourism services [8]. The online portrayal of bariatric tourism services tended to focus on optimistic and positive aspects of previous patient experiences, with only a single negative testimonial available to prospective patients. The ethical problems associated with offering enticements including discounted plastic surgery and of deviating from accepted clinical practice regarding patient selection are well described [9].

Our study was limited to websites published in the English language. It is possible that some bariatric tourism providers do not use the internet to advertise their services to patients and that more conventional doctor-to-doctor referral occurs. It is also conceivable that some websites do not accurately depict the quality of clinical services or patient follow-up provided by their respective bariatric units. Future research should include direct contact with bariatric tourism providers whose services are advertised online to obtain further information about their policies and procedures. Previous research has reflected the often-negative attitudes of healthcare professionals to bariatric surgery tourism [10]. Studies of the lived experiences and attitudes of bariatric tourists themselves have not been done but should be prioritised. We reiterate the call made by others for the creation of an international registry of accredited bariatric tourism providers [11]. Formal efforts by international obesity organisations to enhance public engagement and patient education about bariatric tourism risks and benefits seem warranted.

## Data Availability

The data that support the findings of this study are available from the corresponding author, GTF, upon reasonable request.
